# *Leishmania braziliensis* prostaglandin F_2α_ synthase impacts host infection

**DOI:** 10.1186/s13071-020-3883-z

**Published:** 2020-01-08

**Authors:** Eliza Vanessa Carneiro Alves-Ferreira, Tiago Rodrigues Ferreira, Pegine Walrad, Paul M. Kaye, Angela Kaysel Cruz

**Affiliations:** 1 Department of Cell and Molecular Biology, Ribeirao Preto Medical School, University of Sao Paulo, Ribeirão Prêto, Brazil; 2 Centre for Immunology and Infection, Department of Biology and Hull York Medical School, University of York, York, UK

**Keywords:** *Leishmania braziliensis*, *Lbr*PGF2S, Macrophage, Host-parasite, PGF2α

## Abstract

**Background:**

Prostaglandins (PG) are lipid mediators derived from arachidonic acid metabolism. They are involved in cellular processes such as inflammation and tissue homeostasis. PG production is not restricted to multicellular organisms. Trypanosomatids also synthesize several metabolites of arachidonic acid. Nevertheless, their biological role in these early-branching parasites and their role in host-parasite interaction are not well elucidated. Prostaglandin F_2α_ synthase (PGF2S) has been observed in the *Leishmania braziliensis* secreted proteome and in *L. donovani* extracellular vesicles. Furthermore, we previously reported a positive correlation between *L. braziliensis* PGF2S (*Lbr*PGF2S) expression and pathogenicity in mice.

**Methods:**

*Lbr*PGF2S gene expression and PGF2α synthesis in promastigotes were detected and quantified by western blotting and EIA assay kit, respectively. To investigate *Lbr*PGF2S localization in amastigotes during bone marrow-derived macrophage infection, parasites expressing mCherry-*Lbr*PGF2S were generated and followed by time-lapse imaging for 48 h post-infection. PGF2S homolog sequences from *Leishmania* and humans were analyzed *in silico* using ClustalW on Geneious v6 and EMBOSS Needle.

**Results:**

*Leishmania braziliensis* promastigotes synthesize prostaglandin F_2α_ in the presence of arachidonic acid, with peak production in the stationary growth phase under heat stress. *Lbr*PGF2S is a cytoplasmic protein enriched in the secretory site of the parasite cell body, the flagellar pocket. It is an enzyme constitutively expressed throughout promastigote development, but overexpression of *Lbr*PGF2S leads to an increase of infectivity *in vitro*. The data suggest that *Lbr*PGF2S may be released from intracellular amastigotes into the cytoplasm of bone marrow-derived macrophages over a 48-hour infection period, using time-lapse microscopy and mCherry-PGF2S (mChPGF2S)-expressing parasites.

**Conclusions:**

*Lbr*PGF2S, a parasite-derived protein, is targeted to the host cell cytoplasm. The putative transfer of this enzyme, involved in pro-inflammatory lipid mediator synthesis, to the host cell suggests a potential role in host-parasite interaction and may partially explain the increased pathogenicity associated with overexpression of *Lbr*PGF2S in *L. braziliensis*. Our data provide valuable insights to help understand the importance of parasite-derived lipid mediators in pathogenesis.
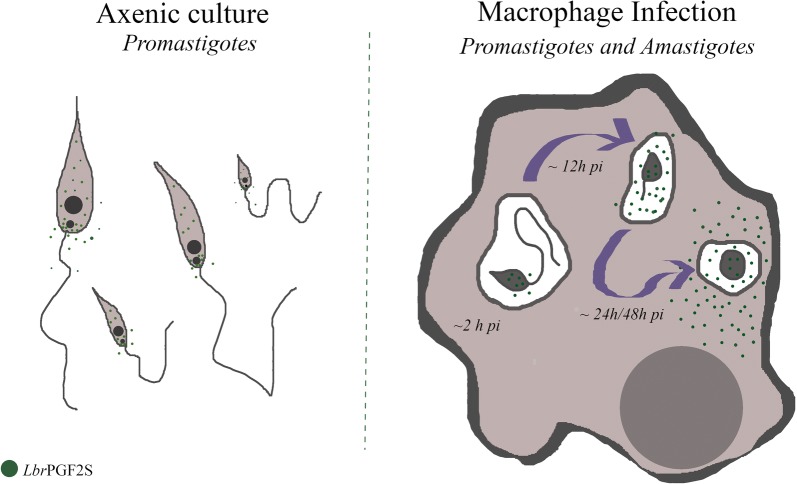

## Background

*Leishmania* (*Viannia*) *braziliensis* is the most virulent agent of localized cutaneous (LCL) and mucocutaneous (MCL) leishmaniases in Brazil [1]. Mucosal commitment occurs in approximately 5–10% of patients infected with *L. braziliensis* [2] and this clinical form is most often diagnosed months or years after the primary clinical manifestation of LCL. Otorhinolaryngological examination of patients from an endemic area in Brazil detected the parasite in the nasal mucosae during early infection, in the absence of mucosal lesions [3]. The mechanisms that facilitate mucosal involvement during *L. braziliensis* infection are poorly understood.

In a previous study, we performed gene expression analyses on two pairs of mucosal and cutaneous *L. braziliensis* isolates, in which proteomic profile differences between isolates were detected [4]. Comparative proteomic analysis revealed a consistently differential pattern of prostaglandin F_2α_ synthase expression (*Lbr*M.31.2410; *Lbr*PGF2S), with higher protein abundance in cutaneous isolates compared to mucosal isolates [4]. Data deposited on TriTrypDB (tritrypdb.org) indicate that *Lbr*PGF2S has been identified in the secretome of *L. braziliensis* and exosomes derived from *Leishmania donovani*. In addition, the Tropical Disease Research Targets Database (tdrtargets.org) indicates that the *Leishmania major* PGF2S (*Lm*PGF2S) homolog has 13 putative antigenic epitopes with 77.8% antigenicity. The structure of *Lm*PGF2S protein has been resolved by crystallization [5] facilitating future study for drug design using this protein.

The synthesis and functions of prostaglandins (PGs) are well characterized mainly in terms of mammalian physiology. In mammals, prostaglandin synthases catalyze the production of prostaglandins using arachidonic acid (AA) metabolites as substrates. AA is removed from membranes by the action of phospholipase A2 and converted into prostaglandin H2 (PGH_2_) by cyclooxygenases (COX-1 or COX-2). PGH_2_ is then converted into several metabolites, such as PGD_2_, PGE_2_ and PGF_2α_, by prostaglandin synthases (e.g. PGF2S) [6]. In mammals, PGF_2α_ is mostly related to ovulation, luteolysis, uterine contraction and the onset of labor [7]. However, it has been recently reported that PG production is not restricted to mammals, occurring in trypanosomatids [6] and other protozoan parasites, such as *Plasmodium falciparum* [8] and *Entamoeba histolytica* [9] [10]. High levels of PGF_2α_ have been detected in *Trypanosoma brucei*, catalyzed by *Tb*PGF2S from PGH_2_ [11]. The prostaglandin F_2α_ synthase activity has been demonstrated for three trypanosomatids orthologous genes. Roberts and colleagues [12] have shown that, in spite of the lack of credible cyclooxygenases, these parasites use arachidonic acid as a substrate to produce PGF_2α_. Additionally, these and other authors demonstrated that some of these kinetoplastid aldo-keto reductases metabolize toxic ketoaldehydes, playing a role as detoxification agents and possibly acting in cellular defense [12, 13]. However, the importance of these pathways for parasite biology and host interaction remains under-explored.

We have shown a positive correlation between *Lbr*PGF2S ectopic overexpression in *L. braziliensis* and the rate of *in vitro* infection [4], suggesting that *Lbr*PGF2S has a role in parasite virulence. In addition, a study on *Leishmania infantum chagasi* showed that PGF2S is highly expressed in metacyclic promastigotes [14]. These authors also observed an increase in lipid bodies (sites for prostaglandin synthesis in mammalian cells) in macrophages infected with *L. infantum*. In our study, we examined PGF_2α_ production in *L. braziliensis*, the expression profile of *Lbr*PGF2S during promastigote development and its localization. Our results are indicative that *Lbr*PGF2S might be transferred from intracellular parasites into the cytoplasm of mouse macrophages. This work, in conjunction with others [4, 14], lend weight to the hypothesis that PGs are parasite virulence factors.

## Methods

### Culture of parasites and infections

Promastigotes of all the *Leishmania braziliensis* wild type strains; BA778 (MHOM/BR/00/BA778), *Lb*2903 (MHOM/BR/75/M2903), H3227 (MHOM/BR/94/H3227), and transfectant strains *Lb*2903[*mChPGF2S::SSU*] and *Lb*2903[*mCherry::SSU*] were cultured at 26 °C in 1× M199 medium supplemented with 0.04 M HEPES, 0.1 mM adenine, 50 µg/ml biotin, 0.25% hemin, 20% FSB, 2.5 U/ml penicillin, 2.5 mg/ml streptomycin and 5 µg/ml biopterin. Transfectants were kept in liquid medium containing G418 (4 µg/ml, 4 × LD_50_).

### Sequence alignment analysis

The protein sequences of *Lbr*PGF2S homologs were obtained from TriTrypDB (http://tritrypdb.org/tritrypdb/). Multiple alignments were performed using ClustalW and Geneious v6 [15] (Biomatters Ltd, Auckland, New Zealand). Global alignment and quantification of identity/similarity were performed using the online version of Needle EMBOSS Needle (ebi.ac.uk/Tools/psa/emboss_needle). To compare the protein 3D structure available in the PDB (http://www.rcsb.org/pdb/) for PGF2S from *L. major* (pdb 4g5d) and AKR1C3 (pdb 4yvv), RCSB’s online Comparison Tool and jFatCat_rigid alignment algorithm were used. Alignments were visualized in Geneious v6.

### SDS-PAGE and immunoblotting analysis

Promastigotes (1 × 10^7^) were harvested from the culture on days 2, 3, 4, 5, 6, 7 and 8, and resuspended in Laemmli sample buffer (500 mM Tris-HCl pH 6.8; 20% glycerol; 0.001% bromophenol blue; 2% SDS; 0.28 M β-mercaptoetanol). To evaluate promastigote secretion, 50 ml of a 7-day-old culture supernatant was collected, filtered through 0.22 µm syringe filters and the proteins precipitated with 10% TCA. Protein extracts were homogenized, denatured at 95 °C for 5 min and loaded on a 12.5% acrylamide gel. Western blotting assays were then performed according to Alves-Ferreira et al. [4].

### Overexpression target construction and transfection

The *Lbr*PGF2S CDS (coding DNA sequence) was amplified from the genomic DNA (gDNA) of *L.* (*V.*) *braziliensis* strain MHOM/BR/75/M2904 using primers *Lbr*PGF2S-nostart-BglII-For (5ʹ-TCA AGA TCT GCT GGG GCC GCT GGG GCC ATC AAC GTT GGT AAG ACC G -3ʹ) and *Lbr*PGF2S-BamHI-Rev (5ʹ-TCA GGA TCC TCA GAA CTG CGC CTC ATC A -3ʹ). The PCR products were digested with *Bgl*II and *Bam*HI enzymes and cloned into the pmCherry-C1 (Addgene, Cambridge, MA, USA) plasmid digested with same enzymes. The mCherry-PGF2S plasmid was then digested with *Pme*I and *Nde*I enzymes. Promastigotes were transfected with mCherry-PGFS or mCherry linear fragments by electroporation [16]. Transfectant colonies were extracted from M199-agar medium in the presence of the G418 antibiotic (Sigma-Aldrich, St. Louis, MO, USA). The G418 LD_50_ was determined for the *Lb*2903 strain, and at four-fold LD_50_ drug concentration (4 µg/ml). Parasites overexpressing *Lbr*PGF2S ectopically were produced and kept as described in [4].

### Bone marrow-derived macrophage (BMDM) production and *in vitro* infection

BMDMs were produced following the protocol described elsewhere [17]. The macrophages were infected with late-stationary-phase *Leishmania* promastigotes (MOI 10:1). For prostaglandin receptor (FP) inhibition, infected macrophages were treated with prostaglandin F_2α_ dimethyl amide (Cayman Chemical, Michigan, USA), an FP receptor antagonist, for up to 24 h or 48 h.

### Immunofluorescence

Promastigotes (in early logarithmic phase) were harvested by centrifugation at 2500× *rpm* for 10 min, washed in PBS and fixed in 2% paraformaldehyde for 10 min. The fixed cells were centrifuged, suspended in 1 M glycine solution and attached to coverslips. Permeabilization was performed using 0.3% Triton X-100 for 10 min and blocking with 2% BSA in PBS for 1 h. We have previously generated an anti-*Lbr*PGF2S chicken IgY antibody by immunizing chickens with His-LbrPGF2S heterologously expressed in *Escherichia coli* [4]. The parasites were then incubated in anti-*Lbr*PGF2S and anti-tubulin (Millipore, Massachusetts, USA) antibodies at 1:10,000 in 1% BSA-PBS for 1 h and washed three times in PBS-T. Secondary anti-chicken conjugated CF488A (Sigma-Aldrich) or anti-mouse conjugated Alexa 555 was added at 1:1000 dilution, supplemented with 60 µM DAPI. Coverslips were washed in PBS-T and MilliQ water and then mounted with ProLong Gold Antifade Reagent (Invitrogen, California, USA). Images were acquired on a Zeiss LSM 510 confocal microscope.

To acquire time lapse images of *in vitro* infection, bone marrow derived macrophages were infected with *Lb*2903[*mChPGF2S::SSU*] and *Lb*2903[*mCherry::SSU*] parasites for 2 h. The cultures were then washed three times with PBS and complete RPMI medium (RPMI-1640, 20% FCS) was added. The culture was kept in the BioStation IMQ (Nikon, Tokio, Japan) incubation chamber at 33 °C and 5% CO_2_ for up to 48 h. The images were captured using a fluorescent field (587 nm excitation and 610 nm emission) and bright field to produce 18 min videos. The images were converted to TIFF format and both images and video processed using ImageJ (Fiji, LOCI, Madison, EUA) and GIMP (GNU Image Manipulation Program).

### Dosage of prostaglandin F_*2α*_

Prostaglandin F_2*α*_ was measured in the supernatant (filtered through 0.22 µm syringe filters) of the *L. braziliensis* culture on growth days 3 and 7 using the immunoenzymatic EIA assay PGF_2α_ KIT (Cayman Chemical, Michigan, USA), according to manufacturer’s instructions.

## Results

### PGF2S ortholog sequences are highly conserved among *Leishmania* spp.

**Fig. 1:**
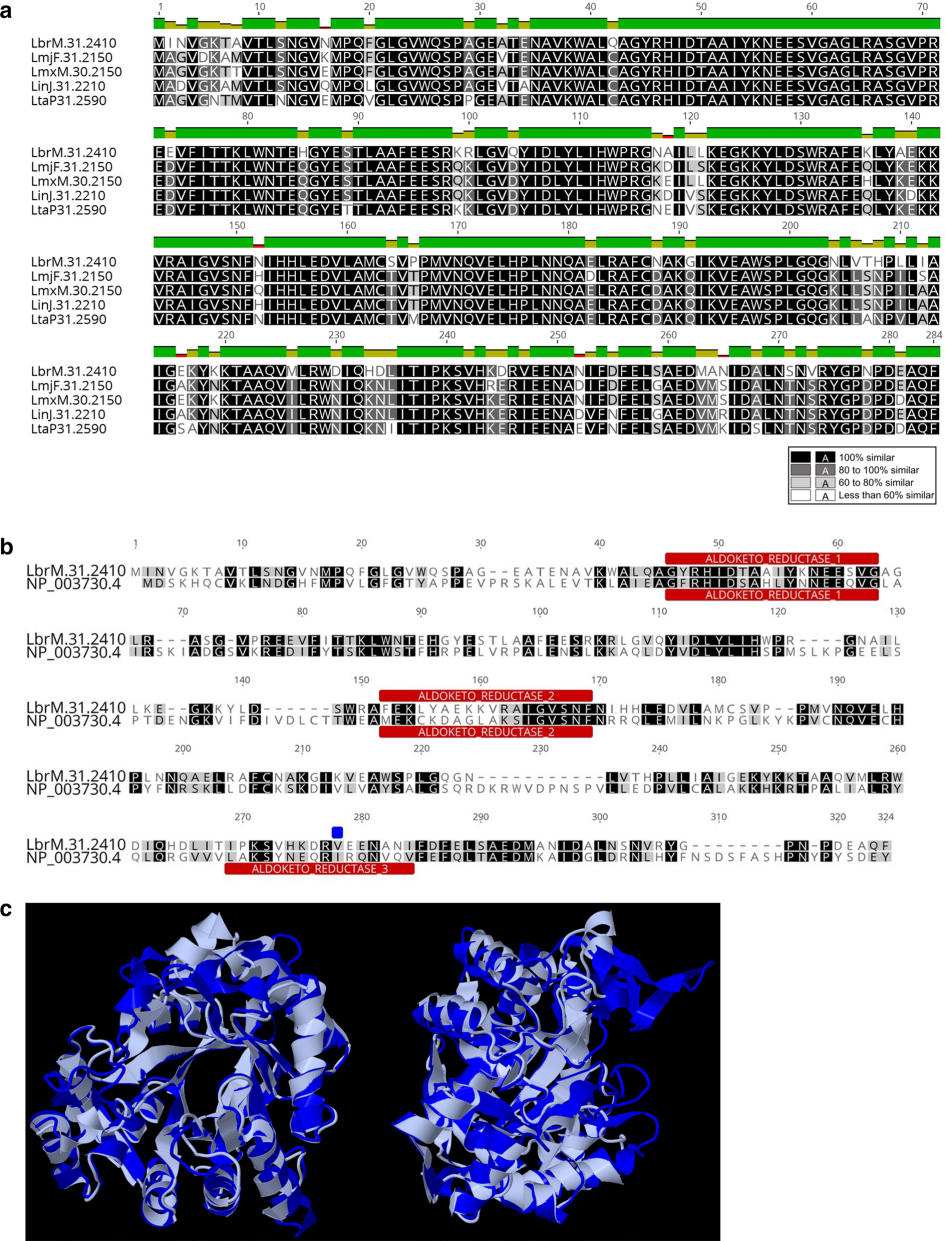
Comparative analysis of the *Lbr*PGF2S amino acid sequence. **a** Multiple sequence alignment of putative PGF2S proteins from *L. braziliensis* (LbrM.31.2410), *L. major* (LmjF.31.2150), *L. mexicana* (LmxM.30.2150*)*, *L. infantum* (LinJ.31.2210) and *L. tarentolae* (LtaP.31.2590). **b** Sequence alignment of *L. braziliensis* PGF2S and human putative ortholog (AKR1C3, NP_003730.4, gi|24497583) using the ClustalW algorithm. Aldo/keto reductase domains are shown in red and the blue square indicates a I247V mutation. **c** 3D sequence alignments of protein sequences of *Lmj*PGF2S (PBD ID 4G5D, in grey) and human ortholog AKR1C3 (PDB ID 4YVV in blue), using RCSB PDB’s online comparison tool (rcsb.org/pdb)

Firstly, we confirmed high sequence homology between prostaglandin F_2α_ synthase genes in *Leishmania* spp. by performing a multiple sequence alignment for *Lbr*PGF2S (LbrM.31.2410) orthologs across different *Leishmania* genomes available in TriTrypDB (tritrypdb.org) (Fig. 1a). We then compared the protein sequence (Fig. 1b) and the 3D structure (Fig. 1c) of *Lbr*PGF2S and the best characterized human PGF2S, AKR1C3 (NP_003730.4), and found 51.4% similarity and 34.3% identity. Additionally*, in silico* analysis of protein domains revealed the presence of two aldo/keto reductase domains (IPR18170) and a putative secretion cleavage signal near the C-terminus in the *Leishmania* PGF2S, suggesting that the protein might undergo proteolysis for subsequent export (Fig. 1b). A third aldo/keto reductase domain was detected in the C-terminal region of AKR1C3, but not in *Lbr*PGF2S, probably due to I247V mutation in this enzyme. PGF2S orthologs in *L. major* (LmjF.31.2150), *L. mexicana* (LmxM.30.2150), *L. infantum* (LinJ.31.2210) and *L. tarentolae* (LtaP.31.2590) carry the predicted third Aldo-Keto domain present in the protein sequences from other organisms. In contrast, PGF2S from *Leishmania braziliensis* (LbrM.31.2410) and all the other *Leishmania* strains from the subgenus *Viannia* (deposited on TriTrypDB) lost the third domain, all of them carry a valine in the isoleucine position, which differs from the strains of the subgenus *Leishmania*.

### Evaluation of *Lbr*PGF2S expression and PGF_2α_ production in promastigotes

**Fig. 2:**
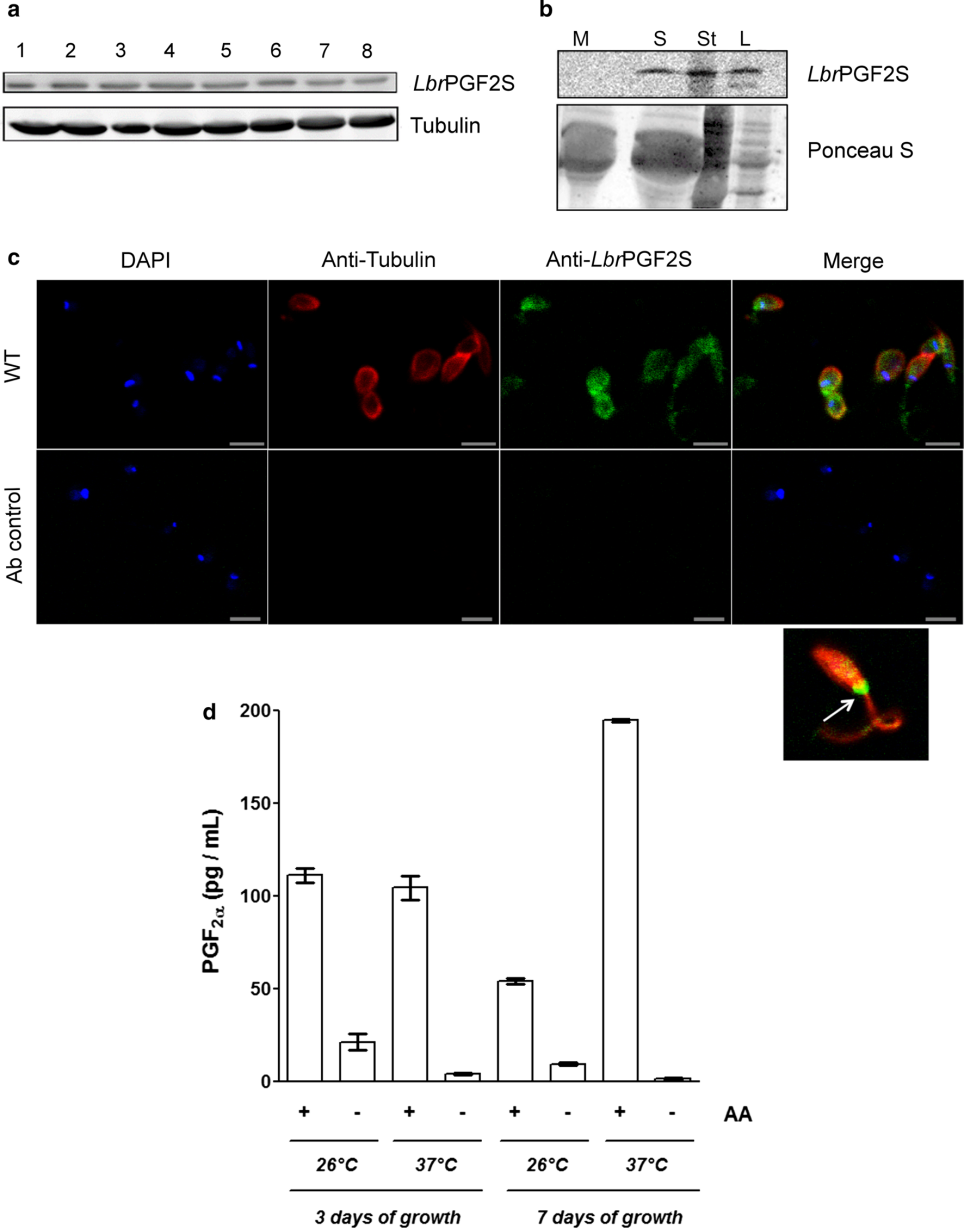
*Lbr*PGF2S expression and secretion in *L. braziliensis* promastigotes. **a** Evaluation of *Lbr*PGF2S protein levels in *L. braziliensis* promastigotes grown in axenic culture for 8 days using polyclonal anti-*Lbr*PGF2S. Anti-Tubulin antibody was used for protein loading control. **b** Secretion of *Lbr*PGF2S by promastigotes in the axenic medium. Immunoblots were used to detect *Lbr*PGF2S in logarithmic (L) and stationary (St) phase promastigotes, 3 and 7 days of culture, respectively, or in the supernatant of the stationary phase culture (S) using anti-*Lbr*PGF2S. Parasite-free M199 medium (M) was used as a negative control. Blots were stained with Ponceau S (lower panel) for protein loading control. **c** Immunofluorescence imaging used to detect *Lbr*PGF2S in wild type promastigotes (at stationary phase). *Lbr*PGF2S (CF488A, green); tubulin (Alexa555, red); DNA was stained with DAPI (blue). An image of a single promastigote is shown bottom right; a strong fluorescence signal appears at the flagellar pocket (white arrow). Ab control: parasites submitted to the same labelling protocol without primary antibodies. **d** Dosage of prostaglandin F_2a_ synthesized by promastigotes in the presence (+) or absence (−) of 66 µM arachidonic acid (AA), measured by EIA assay. Quantification was performed with promastigotes at day 3 (logarithmic phase) or 7 (stationary phase), at 26 °C or 37 °C, as indicated below the x-axis. Data are means ± standard deviation from three replicates. *Scale-bars*: **c**, 5 µm

To evaluate whether *Lbr*PGF2S protein levels are modulated during axenic promastigote differentiation, whole cell extracts were fractionated by SDS-PAGE, and *Lbr*PGF2S analyzed by immunoblotting. No significant changes in PGF2S abundance were observed from day 1 to day 8 (from log to stationary phase, enriched for procyclic and metacyclic forms, respectively), indicating that *LbrPGF2S* is constitutively expressed in promastigotes during *in vitro* growth (Fig. 2a). We then analyzed the presence of *Lbr*PGF2S in the supernatant of stationary promastigote culture (day 7 post-inoculum). Promastigotes were removed by centrifugation and supernatants filtered through a 0.22 μm syringe filter, excluding parasites but not vesicles, such as exosomes. *Lbr*PGF2S was detected by immunoblotting in both parasite lysates and culture supernatant (Fig. 2b). Indirect immunofluorescence using anti-*Lbr*PGF2S antibody revealed cytoplasmic distribution of *Lbr*PGF2S with a noticeably stronger signal near the flagellar pocket (Fig. 2c), a secretory organelle in trypanosomatids. To confirm that axenic *L. braziliensis* promastigotes produce PGF_2α_, the levels of PGF_2α_ in the culture supernatant were quantified at growth days 3 and 7 using an immunoenzymatic assay. Prior to evaluation, the cells were kept at either 26 °C or 37 °C for 4 h in the presence or absence of arachidonic acid (AA). Under all the conditions tested, we observed an increment in PGF_2α_ levels in the supernatant when AA was added. The effect was particularly evident in samples kept at 37 °C (Fig. 2d).

**Fig. 3:**
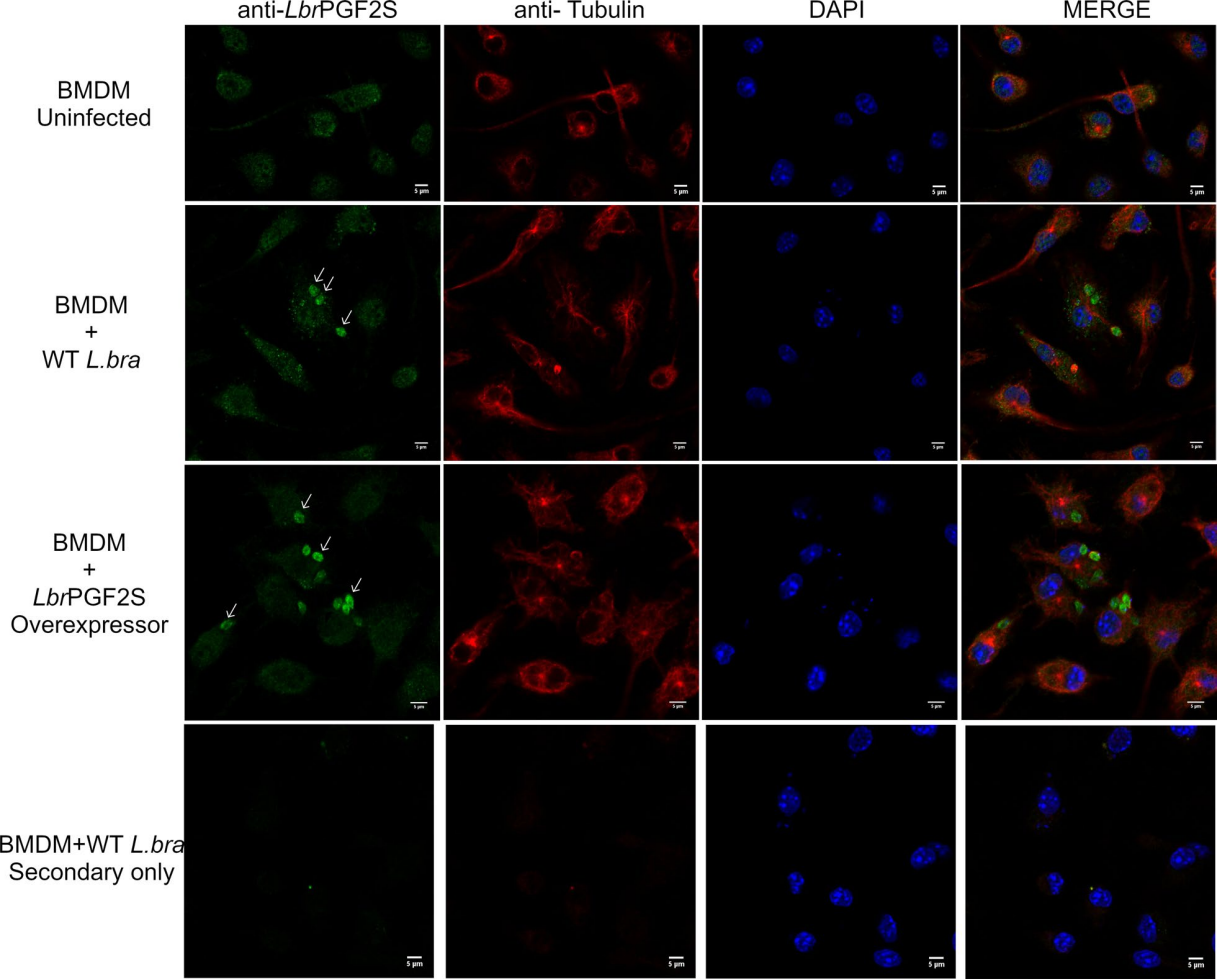
*Lbr*PGF2S detection in *L. braziliensis*-infected macrophages. Immunofluorescence imaging of uninfected bone marrow-derived macrophages (BMDM, top row), macrophages infected with wild type promastigotes (middle row) and *Lbr*PGF2S-overexpressing transfectants (OE, BA778[*pXLbrPGF2S*]) (bottom row), for 48 h. DNA was stained with DAPI (blue); *Lbr*PGF2S (CF488A, green); tubulin (Alexa555, red). *Scale-bars*: 5 μm

Next, to investigate *Lbr*PGF2S expression in amastigotes, mouse bone marrow-derived macrophages (BMDM) were used for *in vitro* infection with wild type (WT) *L. braziliensis* late-stationary phase promastigotes and transfectants overexpressing *Lbr*PGF2S ectopically (*Lb[pXLbrPGF2S]*). After 48 h of infection, *Lbr*PGF2S was detected in WT intracellular amastigotes and at higher levels in macrophages infected with the overexpressing parasites (Fig. 3).

### Detection of *Lbr*PGF2S in host cell cytoplasm

To robustly demonstrate the localization of *Lbr*PGF2S in amastigotes and infected host macrophages in real time during *in vitro* infection, we generated *L. braziliensis* lines expressing *Lbr*PGF2S fused with fluorescent protein mCherry (mChPGF2S). These constructs overcome the problem associated with cross-reaction of the *Lbr*PGF2S antibody with macrophage molecules. Plasmids pSSU-Neo-mCherry (control) and pSSU-Neo-mChPGF2S were used to generate lines *Lb*2903[*mCherry::SSU*] and *Lb*2903[*mChPGF2S::SSU*] (hereinafter referred as *Lb*[mCherry] and *Lb*[mChPGF2S], respectively) (Additional file 1: Figure S1a). Correct integration of mCherry constructs in the ribosomal locus was confirmed by PCR (Additional file 1: Figure S1b). We then confirmed the presence of both the SSU-integrated mChPGF2S (~61kDa) and the endogenous *Lbr*PGF2S (~31 kDa) using anti-*Lbr*PGF2S antibody (Additional file 1: Figure S1c).

Similarly to parasites overexpressing wild type *Lbr*PGF2S ectopically [4], *Lb*[mChPGF2S] stationary phase promastigotes infected BMDMs more effectively than *Lb*[mCherry], the control parasites (Additional file 2: Figure S2). To investigate the impact of the PGF2α pathway on the *L. braziliensis in vitro* infection we performed an inhibition assay by treating infected BMDM with an FP receptor antagonist for 24 h (prostaglandin F2α dimethyl amide). FP inhibition decreases the percentage of infected macrophages with *L. braziliensis* wild type (Strain H3227) (a 10-fold decrease at 10 µg/ml) and the number of amastigotes per cell (a 18.5-fold decrease at 10 µg/ml) (Additional file 3: Figure S3).

**Fig. 4:**
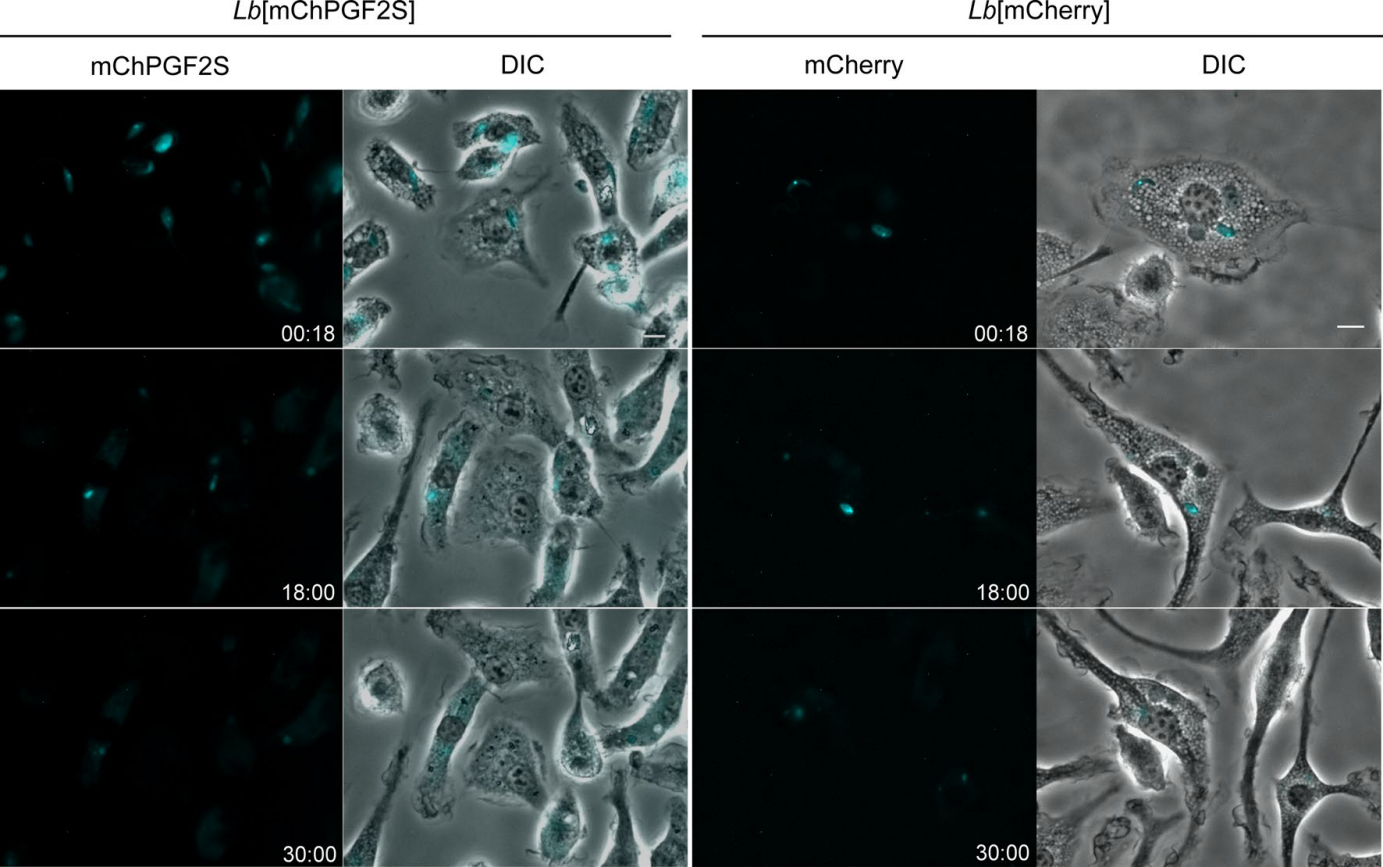
Parasite-derived mChPGF2S accesses the host cell cytoplasm. Time-lapse images were captured from mouse bone marrow-derived macrophages infected with *Lb*[mCherry] and *Lb*[mChPGF2S] promastigotes, as indicated in the figure. Cyan was used as a pseudocolor; numbers at the bottom right of each panel indicate hour:minutes after infection. See videos in Additional files 4, 5, 6, 7, 8, 9. *Scale-bar*: 5 µm

**Fig. 5:**
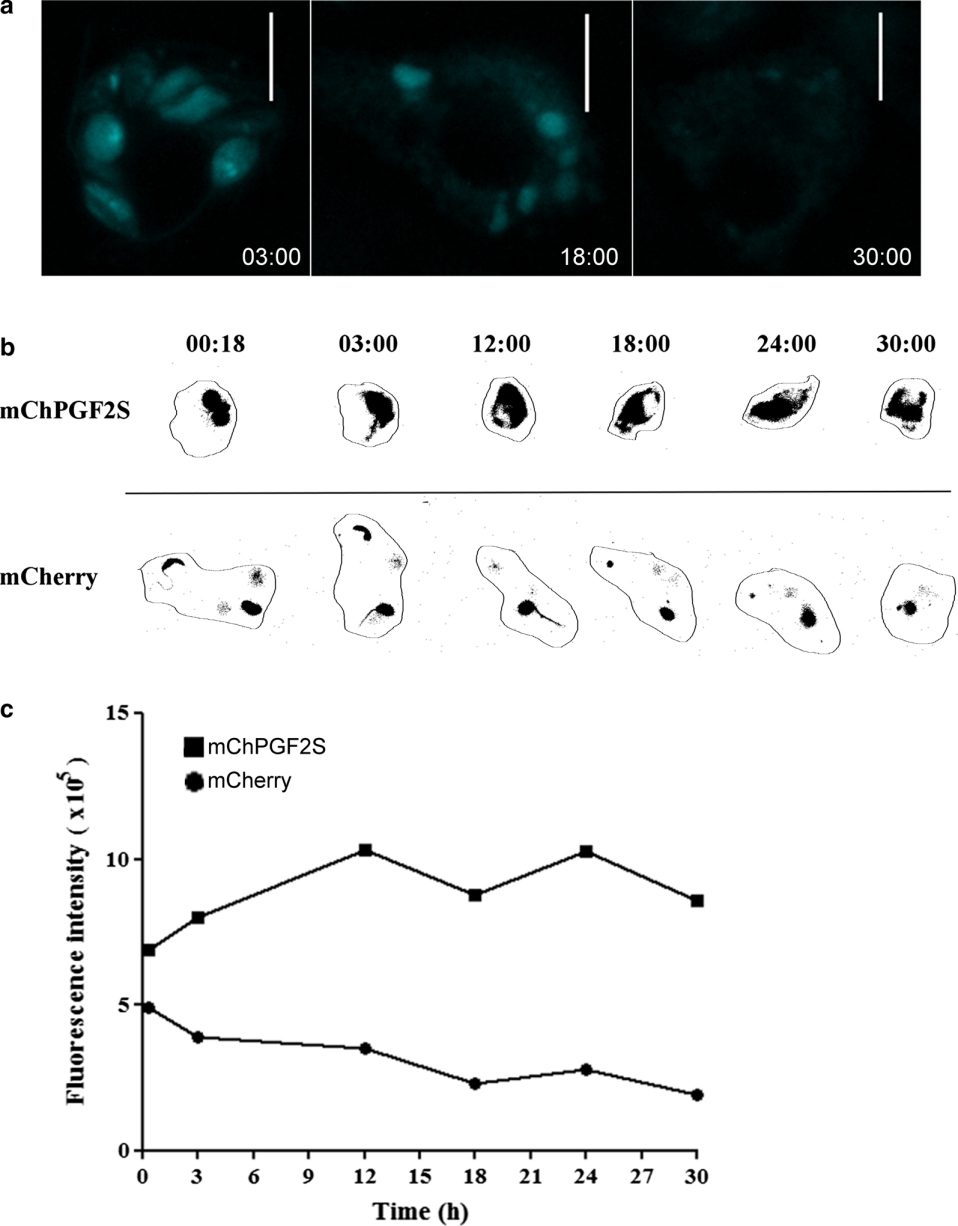
Detection of mChPGF2S in highly-infected macrophages. **a** Detection of mCherry fluorescence in the host cell cytoplasm. Time-lapse images of macrophages infected with more than three mChPGF2S parasites. Cyan was used as pseudocolor; numbers at the bottom right of each panel indicate hour:minutes after infection. **b** Close image analysis of mCherry and mChPGF2S localized fluorescence intensity in infected macrophages. Numbers at the top of each image indicate hour:minutes after infection. **c** Overall intracellular fluorescence in infected macrophages was quantified using FIJI (ImageJ) software up to 30 h pi. *Scale-bars*: **a**, 10 µm

To investigate *Lbr*PGF2S distribution, macrophages were infected with late stationary phase promastigotes from *Lb*[mCherry] and *Lb*[mChPGF2S] parasites, followed by time-lapse imaging for 48 h. Fluorescence of mChPGF2S was detected mainly inside intracellular promastigotes in the early stages of infection. Surprisingly, at ~18 h post-infection (pi), mChPGF2S was observed in the macrophage cytoplasm and subsequently found dispersed. Note that at this time the parasites had not fully differentiated into amastigotes, although most gene expression changes take place in this initial phase of differentiation [18]. This staining pattern was not observed in control *Lb*[mCherry] intracellular parasites (Fig. 4, Additional file 4: Video S1, Additional file 5: Video S2, Additional file 6: Video S3, Additional file 7: Video S4). The distribution of mChPGF2S fluorescence throughout the host cell cytoplasm was more clearly observed in macrophages with a higher number of intracellular parasites (Fig. 5a, Additional file 8: Video S5, Additional file 9: Video S6). Nevertheless, even macrophages infected with only two mChPGF2S parasites exhibited dispersion of the fluorescence around the entire cell body, while fluorescence in macrophages infected with mCherry parasites was restricted to the vacuole region (promastigotes and amastigotes) (Fig. 5b).

## Discussion

This study shows that *Lbr*PGF2S, which might contribute to *Leishmania* virulence profiles in the mammalian host [4], is found in host macrophage cytoplasm infected with *L. braziliensis,* suggesting direct interaction with the host cell. In addition, *Lbr*PGF2S expression was also detected in *L. braziliensis* promastigotes in axenic culture throughout promastigote growth and in the supernatant. The protein might be secreted through the flagellar pocket, as indicated by the strong signal detected under immunofluorescence. These results corroborate and extend previous studies that have identified PGF2S in the secreted proteome of *L. braziliensis* [19].

Although PGF_2α_ synthesis by recombinant *Lbr*PGFS was not measured herein, other researchers have shown that PGF2S homologs in *L. major*, *L. tropica*, *L. donovani*, *L. infantum*, *T. cruzi* and *T. brucei* catalyze PGF_2α_ synthesis *in vitro* [6, 12]. Since we detected the production of PGF_2α_ by promastigotes in axenic culture, we suggest that *Lbr*PGF2S has the same catalytic function. To the best of our knowledge, we are the first, however, to observe that *Lbr*PGF2S is expressed in amastigotes and localized in the host cell cytoplasm infected with *Leishmania*. Other authors have shown that vesicles released by *Leishmania* during *in vitro* or *in vivo* infection interfere with host cell response [20]. Recently, it has been shown that promastigotes secret exosomes into the midgut lumen of the sand fly vector and these extracellular vesicles are regurgitated with parasites into the skin during blood meal, modulating the immune response to *Leishmania* infection. Interestingly, the PGF2S protein was found among the 124 proteins identified by proteomic analysis in vesicles from *L. infantum*-infected midguts [21]. Although other experiments are needed to confirm *Lbr*PGF2S secretion, our results are indicative of the transfer of the enzyme into the cytoplasm of infected macrophages. Other proteins secreted to the host cell cytoplasm, such as gp63, were shown to modulate phagocytic cell response [22, 23]. The process by which PGF2S reaches the cytoplasm of the host cell remains unknown, but we have shown that inhibition of the prostaglandin receptor affected negatively the infection profile of BMDM *in vitro*.

Although most studies on PGF_2α_ are related to mammalian female reproduction [7, 24], their role in leukocyte migration has been shown, with high potential to act as a neutrophil chemoattractant *in vitro* [25] and *in vivo* [26]. In addition, AKR1C3 is abundant in keratinocytes in the suprabasal layer of the epidermis and regulates the synthesis of PGF_2α_ in the presence of calcium, contributing to the pro-inflammatory response *in vitro* [27]. Although we have shown that *Lbr*PGF2S and AKR1C3 protein sequences share 51.4% similarity and 34.3% identity, modelling using the Research Collaboratory for Structural Bioinformatics (RCSB) PDB suggests that the protein structures of *Lbr*PGF2S and AKR1C3 are quite similar, sharing at least two aldo/keto reductase domains and indicating that both proteins may retain the core function of PGF_2α_ production. Thus, the work of other researchers using different *Leishmania* species [6, 14] and our own data suggest that the PGF_2α_ signaling pathway could be involved in *Leishmania* pathogenicity.

## Conclusions

To the best of our knowledge, this is the first study to report that *L. braziliensis* promastigotes and amastigotes can express *Lbr*PGF2S and that it is possibly found in the infected host cell cytoplasm. Based on our results and those in the literature, we propose that the production of PGF2S improves the fitness of the parasite and might play a role in the mammalian host-parasite interaction. *Lbr*PGF2S could be a new *Leishmania* virulence factor and is, therefore, a potentially attractive target for drug discovery or vaccine development.

## Supplementary information

**Additional file 1: Figure S1.** Generation and characterization of *L. braziliensis Lbr*PGF2S overexpressor. **a** Targeting fragments for integration of mCherry or mChPGF2S into the ribosomal locus. **b** Primers NEOend-for and SSUdown-rev were used to confirm the expected integration. *Lbr*[mCherry]: mCh 1 and 2; *Lbr*[mChPGF2S]: PG 1 and 2. Negative controls: *Lb*2903 and H3227 gDNA and a PCR without DNA (C-). Positive controls: *Lmj* SSU-NEO transfectant (C+). **c** Immunoblotting using anti-*Lbr*PGF2S antibody. The 61 kDa and 31 kDa bands are mChPGF2S and the endogenous *Lbr*PGF2S, respectively.

**Additional file 2: Figure S2.** *In vitro* infection of BMDMs from BALB/c mice with *Lb*[*mCherry*] and *Lb*[*mChPGF2S*] promastigotes between 0 and 48 h post-infection. **a** Percent of infected macrophages. **b** Number of parasites per 100 macrophages. Results are average ± SD from three replicates. *P < 0.05, **P < 0.01 (ANOVA).

**Additional file 3: Figure S3.** *In vitro* infection of BMDMs with parasites in presence or absence of FP receptor antagonist. Upper panel: percentage of infected macrophages. Lower panel: number of parasites per 100 macrophages, with or without FP receptor antagonist (0, 5 or 10 μg/ml). Results are average ± se from three replicates. Prostaglandin F2α dimethyl amide was used at 5 μg/ml or 10 μg/ml. *P < 0.001 (ANOVA).

**Additional file 4: Video S1.** Macrophages infected with *Lb*[mChPGF2S] parasites.

**Additional file 5: Video S2.** Merge of fluorescent and bright field images of macrophages infected by *Lb*[mChPGF2S] parasites.

**Additional file 6: Video S3.** Macrophages infected with *Lb*[mCherry] parasites.

**Additional file 7: Video S4.** Merge of fluorescent and bright field images of macrophages infected with mCherry parasites.

**Additional file 8: Video S5.** Macrophages infected with a high number of mChPGF2S parasites.

**Additional file 9: Video S6.** Merge of fluorescent and bright field images of macrophages infected with a high number of mChPGF2S parasites.

## Data Availability

Data supporting the conclusions of this article are available in the article and its additional files.

## Abbreviations

LCL: localized cutaneous leishmaniasis
MCL: mucocutaneous leishmaniasis
PGs: prostaglandins
PGF2S: prostaglandin F_2α_ synthase
*Lbr*PGF2S: prostaglandin F_2α_ synthase from *Leishmania braziliensis*
AA: arachidonic acid
PGF_2α_: prostaglandin F_2α_
PGH_2_: prostaglandin H2
AKR1C3: human aldo-keto reductase family 1, member C3
COX-1 or COX-2: cyclooxygenases 1 or 2
CDS: coding sequence
PBS: phosphate-buffered saline
FP: prostaglandin F receptor
18S: 18S ribosomal RNA
5ʹSSU: 5ʹ untranslated region of small ribosomal subunit
3ʹSSU: 3ʹ untranslated region of SSU
CPB\IR: intergenic region of cysteine peptidase B gene
SAS: splice acceptor site
NEO: neomycin phosphotransferase
SD: standard deviation

## References

[1] Barral A, Pedral-Sampaio D, Grimaldi Junior G, Momen H, McMahon-Pratt D, Ribeiro de Jesus A (1991). Leishmaniasis in Bahia, Brazil: evidence that *Leishmania amazonensis* produces a wide spectrum of clinical disease. Am J Trop Med Hyg..

[2] Amato VS, Tuon FF, Bacha HA, Neto VA, Nicodemo AC (2008). Mucosal leishmaniasis. Current scenario and prospects for treatment. Acta Trop..

[3] Boaventura VS, Cafe V, Costa J, Oliveira F, Bafica A, Rosato A (2006). Concomitant early mucosal and cutaneous leishmaniasis in Brazil. Am J Trop Med Hyg..

[4] Alves-Ferreira EV, Toledo JS, De Oliveira AH, Ferreira TR, Ruy PC, Pinzan CF (2015). Differential gene expression and infection profiles of cutaneous and mucosal *Leishmania braziliensis* isolates from the same patient. PLoS Negl Trop Dis..

[5] Moen SO, Fairman JW, Barnes SR, Sullivan A, Nakazawa-Hewitt S, Van Voorhis WC (2015). Structures of prostaglandin F synthase from the protozoa *Leishmania major* and *Trypanosoma cruzi* with NADP. Acta Crystallogr F Struct Biol Commun..

[6] Kubata BK, Duszenko M, Martin KS, Urade Y (2007). Molecular basis for prostaglandin production in hosts and parasites. Trends Parasitol..

[7] Sugimoto Y, Yamasaki A, Segi E, Tsuboi K, Aze Y, Nishimura T (1997). Failure of parturition in mice lacking the prostaglandin F receptor. Science..

[8] Kilunga Kubata B, Eguchi N, Urade Y, Yamashita K, Mitamura T, Tai K (1998). *Plasmodium falciparum* produces prostaglandins that are pyrogenic, somnogenic, and immunosuppressive substances in humans. J Exp Med..

[9] Belley A, Chadee K (2000). Production of prostaglandin E(2) by *Entamoeba histolytica via* a novel cyclooxygenase. Arch Med Res..

[10] Lejeune M, Moreau F, Chadee K (2011). Prostaglandin E2 produced by *Entamoeba histolytica* signals *via* EP4 receptor and alters claudin-4 to increase ion permeability of tight junctions. Am J Pathol..

[11] Kubata BK, Duszenko M, Kabututu Z, Rawer M, Szallies A, Fujimori K (2000). Identification of a novel prostaglandin f(2alpha) synthase in *Trypanosoma brucei*. J Exp Med..

[12] Roberts AJ, Dunne J, Scullion P, Norval S, Fairlamb AH (2018). A role for trypanosomatid aldo-keto reductases in methylglyoxal, prostaglandin and isoprostane metabolism. Biochem J..

[13] Rath J, Gowri VS, Chauhan SC, Padmanabhan PK, Srinivasan N, Madhubala R (2009). A glutathione-specific aldose reductase of *Leishmania donovani* and its potential implications for methylglyoxal detoxification pathway. Gene..

[14] Araujo-Santos T, Rodriguez NE, Moura-Pontes S, Dixt UG, Abanades DR, Bozza PT (2014). Role of prostaglandin F2alpha production in lipid bodies from *Leishmania infantum chagasi*: insights on virulence. J Infect Dis..

[15] Kearse M, Moir R, Wilson A, Stones-Havas S, Cheung M, Sturrock S (2012). Geneious Basic: an integrated and extendable desktop software platform for the organization and analysis of sequence data. Bioinformatics..

[16] Cruz A, Beverley SM (1990). Gene replacement in parasitic protozoa. Nature..

[17] Marim FM, Silveira TN, Lima DS, Zamboni DS (2010). A method for generation of bone marrow-derived macrophages from cryopreserved mouse bone marrow cells. PLoS ONE..

[18] Dillon LA, Okrah K, Hughitt VK, Suresh R, Li Y, Fernandes MC (2015). Transcriptomic profiling of gene expression and RNA processing during *Leishmania major* differentiation. Nucleic Acids Res..

[19] Cuervo P, De Jesus JB, Saboia-Vahia L, Mendonça-Lima L, Domont GB, Cupolillo E (2009). Proteomic characterization of the released/secreted proteins of *Leishmania* (*Viannia*) *braziliensis* promastigotes. J Proteomics..

[20] Silverman JM, Reiner NE (2012). *Leishmania* exosomes deliver preemptive strikes to create an environment permissive for early infection. Front Cell Infect Microbiol..

[21] Atayde VD, Aslan H, Townsend S, Hassani K, Kamhawi S, Olivier M (2015). Exosome secretion by the parasitic protozoan *Leishmania* within the sand fly midgut. Cell Rep..

[22] Brittingham A, Morrison CJ, McMaster WR, McGwire BS, Chang KP, Mosser DM (1995). Role of the *Leishmania* surface protease gp63 in complement fixation, cell adhesion, and resistance to complement-mediated lysis. J Immunol..

[23] Olivier M, Atayde VD, Isnard A, Hassani K, Shio MT (2012). *Leishmania* virulence factors: focus on the metalloprotease GP63. Microbes Infect.

[24] Saito O, Guan Y, Qi Z, Davis LS, Komhoff M, Sugimoto Y (2003). Expression of the prostaglandin F receptor (FP) gene along the mouse genitourinary tract. Am J Physiol Renal Physiol..

[25] Arnould T, Thibaut-Vercruyssen R, Bouaziz N, Dieu M, Remacle J, Michiels C (2001). PGF(2alpha), a prostanoid released by endothelial cells activated by hypoxia, is a chemoattractant candidate for neutrophil recruitment. Am J Pathol..

[26] de Menezes GB, dos Reis WG, Santos JM, Duarte ID, de Francischi JN (2005). Inhibition of prostaglandin F(2alpha) by selective cyclooxygenase 2 inhibitors accounts for reduced rat leukocyte migration. Inflammation..

[27] Mantel A, Carpenter-Mendini AB, Vanbuskirk JB, De Benedetto A, Beck LA, Pentland AP (2012). Aldo-keto reductase 1C3 is expressed in differentiated human epidermis, affects keratinocyte differentiation, and is upregulated in atopic dermatitis. J Investig Dermatol..

