# Supplementary figures and images for: Leishmania braziliensis prostaglandin F2α synthase impacts host infection
Source: Parasit Vectors. 2020 Jan 8;13:9. doi: 10.1186/s13071-020-3883-z (PMC6950890; doi:10.1186/s13071-020-3883-z)

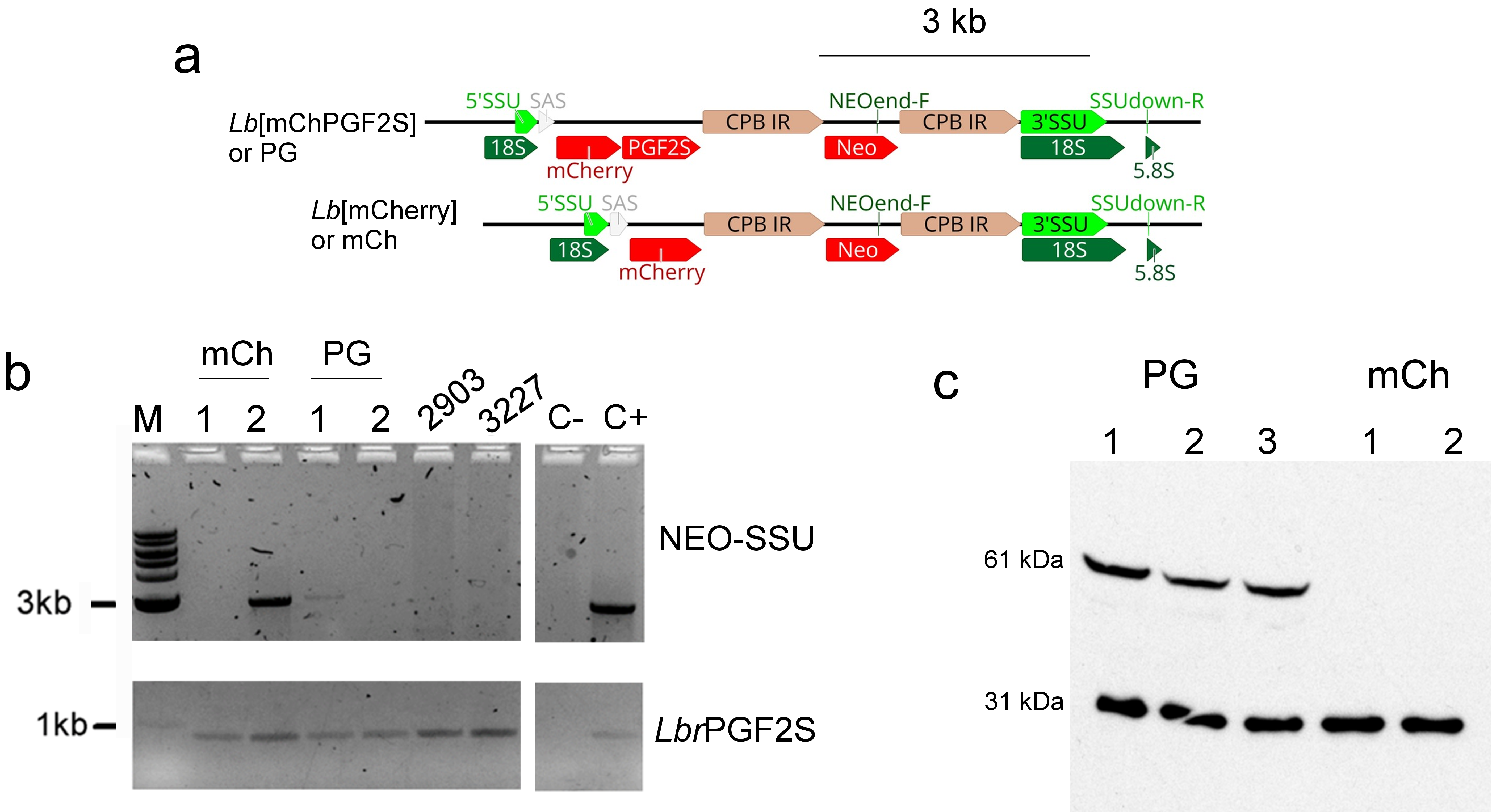

Supplement: Supplementary file 1 — Additional file 1: Figure S1. Generation and characterization of L. braziliensis LbrPGF2S overexpressor. a Targeting fragments for integration of mCherry or mChPGF2S into the ribosomal locus. b Primers NEOend-for and SSUdown-rev were used to confirm the expected integration. Lbr[mCherry]: mCh 1 and 2; Lbr[mChPGF2S]: PG 1 and 2. Negative controls: Lb2903 and H3227 gDNA and a PCR without DNA (C-). Positive controls: Lmj SSU-NEO transfectant (C+). c Immunoblotting using anti-LbrPGF2S antibody. The 61 kDa and 31 kDa bands are mChPGF2S and the endogenous LbrPGF2S, respectively. [file 13071_2020_3883_MOESM1_ESM.tiff]

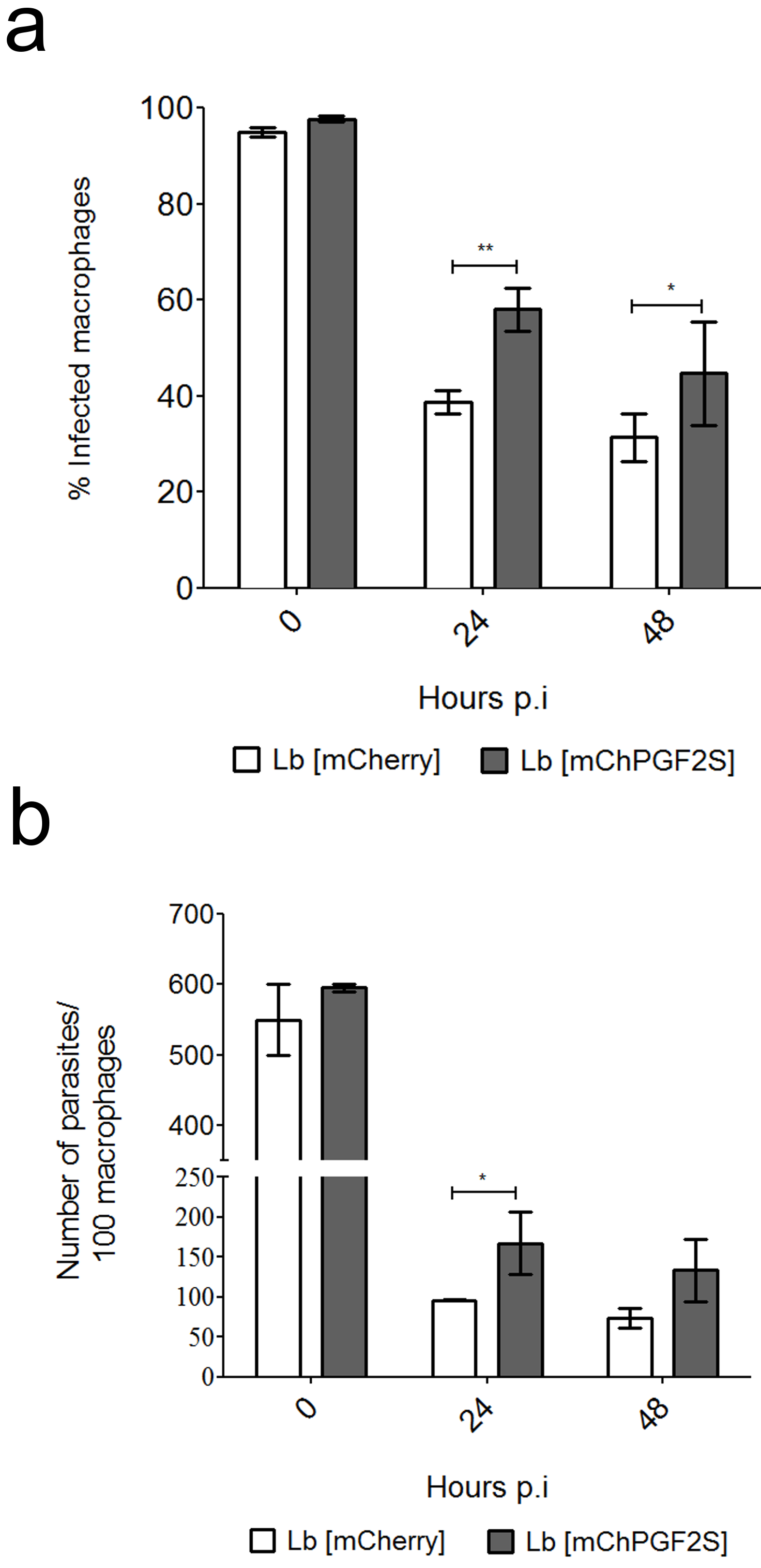

Supplement: Supplementary file 2 — Additional file 2: Figure S2. In vitro infection of BMDMs from BALB/c mice with Lb[mCherry] and Lb[mChPGF2S] promastigotes between 0 and 48 h post-infection. a Percent of infected macrophages. b Number of parasites per 100 macrophages. Results are average ± SD from three replicates. *P < 0.05, **P < 0.01 (ANOVA). [file 13071_2020_3883_MOESM2_ESM.tiff]

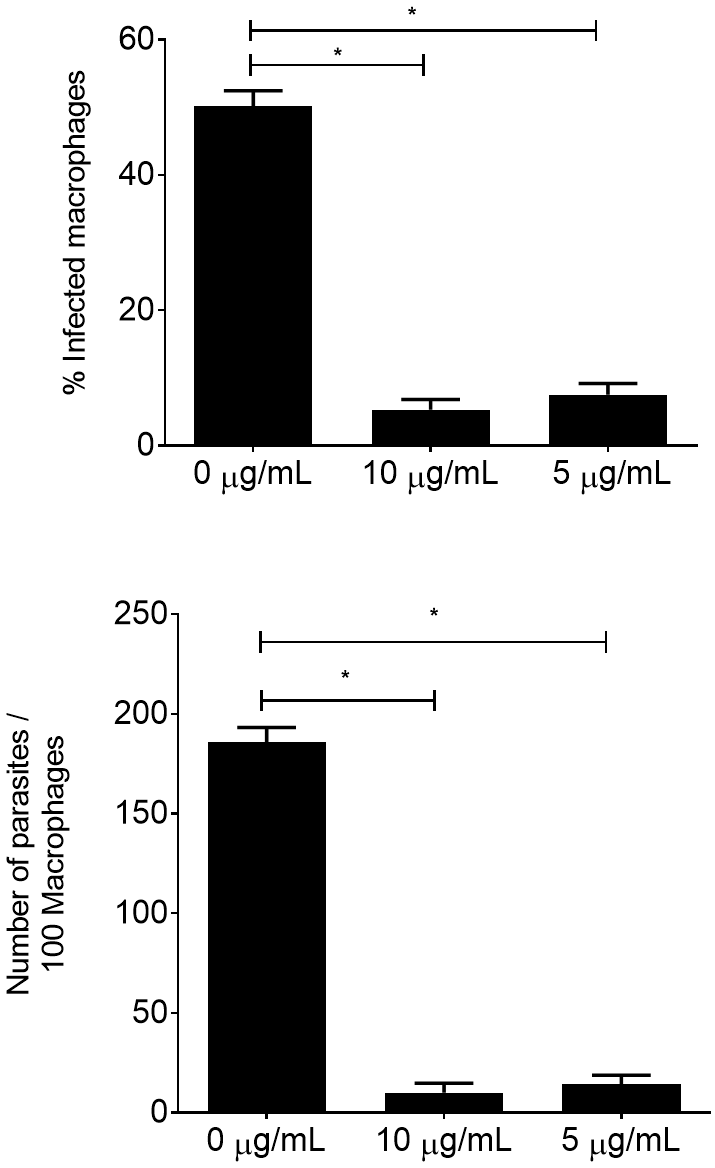

Supplement: Supplementary file 3 — Additional file 3: Figure S3. In vitro infection of BMDMs with parasites in presence or absence of FP receptor antagonist. Upper panel: percentage of infected macrophages. Lower panel: number of parasites per 100 macrophages, with or without FP receptor antagonist (0, 5 or 10 μg/ml). Results are average ± se from three replicates. Prostaglandin F2α dimethyl amide was used at 5 μg/ml or 10 μg/ml. *P < 0.001 (ANOVA). [file 13071_2020_3883_MOESM3_ESM.tiff]
